# General and Specific Combining Abilities for Egg Production Traits in a Two‐Way Cross Between South African Local and Commercial Chicken Breeds

**DOI:** 10.1155/vmi/9981706

**Published:** 2026-07-29

**Authors:** Kwena Mokoena, Vusi Mbazima, Louis Tyasi

**Affiliations:** ^1^ Department of Agricultural Economics and Animal Production, School of Agricultural and Environmental Sciences, University of Limpopo, Private Bag X1106, Sovenga 0727, Limpopo, South Africa, ul.ac.za; ^2^ School of Molecular and Life Sciences, Department of Biochemistry Microbiology and Biotechnology, University of Limpopo, Private Bag X1106, Sovenga 0727, Limpopo, South Africa, ul.ac.za

**Keywords:** age at first egg, combining abilities, crossbreeding, egg weight, hen housed production

## Abstract

This study aimed to determine the general combining ability (GCA) and specific combining ability (SCA) for egg production in a two‐way cross between Potchefstroom Koekoek (PK) and Lohmann Brown (LB) chicken breeds. The study used 352 chickens in a completely randomized design. A total of 524 eggs were collected from the pure and crossbred chickens. *R* (Version 4.4.2) was used for data analysis. The results showed that the GCA effects were highly significant (*p* < 0.01) for age at first egg (0.50) and hen housed production (5.35), and SCA was highly significant (*p* < 0.01) for age at first egg (16.00) and hen housed production (36.49). GCA and SCA had no significant (*p* > 0.05) effect on external and internal egg quality traits. The GCA/SCA ratio results indicated that nonadditive influences (*p* < 0.05) body weight (−0.45) and egg weight (−0.92), while additive influences (*p* < 0.05) albumen ratio (5.89). The study concluded that selection for breeding, prioritizing parents with superior GCA, ensures predictable body weight. The results of the GCA/SCA ratio highlight selection and crossbreeding as ideal for improving body weight and egg weight. Furthermore, the use of a LB sire and PK dam is considered for developing a new strain.

## 1. Introduction

Indigenous chickens are among the poultry that contribute to the livelihood of rural communities in developing countries such as South Africa [1]. Potchefstroom Koekoek (PK) is among the South African recognized indigenous chickens alongside Naked Neck, Ovambo, Boschveld, Venda, and nondescript village chickens [2]. PK is a dual‐purpose breed developed in South Africa for both meat and egg production, laying brown eggs with 55.7 g average weight [3]. Indigenous chickens have the genetic potential to adapt to local climatic conditions and are robust against stressors [4]. However, indigenous chickens are poor in egg production performance; this is due to factors such as slow growth, late sexual maturity, and broodiness for an extended period [5]. The common practice in the poultry industry is to crossbreed chickens to improve the desirable traits through mating of individuals from different genetic backgrounds [6]. PK has been reported to have moderate to high heritability for traits such as egg production, growth traits, and feather color from its parents [7]. The crossbreeding could improve the egg production by exploiting the general combining ability (GCA) (additive) and specific combining ability (SCA) (nonadditive) of genetic variation due to heterosis or hybrid vigor [8, 9]. GCA is defined as the average performance of a line (strain or breed) in hybrid combination with other lines [10], while the SCA measures the average inferiority or superiority of crosses relative to the average performance of their parental breeds [11]. There have been several studies performed on the crossbreeding of chickens [12–15]. The indigenous chickens are often crossed to improve the performance of traits such as egg‐laying performance [16], growth rate [17, 18], disease resistance [19], and feed efficiency [20], thus contributing to income generation for the rural community and food security. Although there have been studies performed on the crossbreeding of chickens, to our knowledge, there are limited studies on the crossbreeding of South African indigenous and commercial chickens to improve egg production. Hence, the objective of the study was to determine the GCA and SCA for egg production in a two‐way cross between Potchefstroom Koekoek (PK) and Lohmann Brown (LB) chicken breeds.

## 2. Materials and Methods

### 2.1. Ethical Approval

The study was submitted for ethical approval before the commencement of the study to the Animal Research and Ethics Committee (AREC) . It was approved under AREC, and the certificate number is AREC/53/2023: PG.

### 2.2. Study Site

The study was carried out at the University of Limpopo Experimental farm, located in the province of Limpopo, South Africa. The ambient temperature fluctuates between 10°C and 25°C in winter and 20°C and 36°C in the summer. The farm is located at 27.55°S and 24.77°E longitude [21].

### 2.3. Preparation of the House

To disinfect the experimental housing and hatchery, Jeyes fluid was used for thorough cleaning with water. To disrupt the life cycle of any disease‐causing agents that may not have been killed by the disinfectant, the house was allowed to dry for 7 days before use. The incubator was properly cleaned and disinfected, as well as every piece of equipment, including feeders and drinkers. A fresh disinfectant was added each day to the footbath.

### 2.4. Chicken Management

A semiopen house with litter floor was used to house the chicks, and it was partitioned according to their breed and crossbred. The crossbreeding experiments took place from April to December 2024. All experimental chickens were subjected to the same climatic condition and vaccinated against Newcastle disease (HB1 and Lasota) at Week 1 and Gumboro (infectious) at Weeks 2 and 3, and vaccines are added into drinking water. The chickens were fed 20% CP and 3000 kcal/kg ME from day old to Week 8; they were then fed a commercial grower ration of 18% CP and 2950 kcal/kg ME from Week 9 to Week 14. The chickens were then fed commercial laying ration (16.5% and 2700 kca/kg). A continuous light program was used to provide artificial heat (32°C) using infrared lights for a period of 24 h in the first week and then was reduced to 18–20 h of lighting until the chicks were 8 weeks old. The artificial light was then withdrawn, and the chickens were allowed 12 h of natural light. Curtain rails were used to regulate ventilation, and commercial feed and water were provided *ad libitum.*

### 2.5. Experimental Chickens and Design

A total of 352 chickens were obtained by mating PK and LB chicken breeds in a 2 × 2 diallel cross experimental design. Ten hens (*n* = 10) from each breed were assigned randomly to be mated with two roosters (*n* = 2) of each breed (Table 1). The experiment was replicated three times.

**TABLE 1:** The 2 × 2 diallel mating design.

Male (♂)			

		**PK (2 roosters)**	**LB (2 roosters)**

Female (♀)	PK (10 hens)	PK × PK	PK × LB
	LB (10 hens)	LB × PK	LB × LB

*Note:* PK × PK, Potchefstroom Koekoek crossed with Potchefstroom Koekoek; LB × PK, Potchefstroom Koekoek crossed with Lohmann Brown; LB × LB, Lohmann Brown crossed with Lohmann Brown.

Abbreviations: LB, Lohmann Brown; PK, Potchefstroom Koekoek.

### 2.6. Data Collection

The individual body weight (BW) of 352 chickens was recorded once at 23 weeks of age. A total of 524 eggs were collected from pure with LB (150 eggs) and PK (129 eggs) and crossbred chicken with LB × PK (119 eggs) and PK × LB (77 eggs). The age at first egg (AFE), hen housed production (HHP), and external and internal egg quality traits were recorded as described by Dzungwe et al. [6] and Hailemariam et al. [18]. The following egg quality traits were measured: Egg weight (EW), egg length (EL), egg diameter (ED), shell weight (SW), shape index (SPI), shell surface area (SS), unit surface area (US), shell ratio (SR), and egg volume (EV) as external traits and albumen weight (AW), yolk weight (YW), albumen ratio (AR), yolk ratio (YR), and YW/AW (Y/A) as internal traits.

### 2.7. Data Analysis

The data were analyzed using the general linear model (GLM) procedure of R (Version 4.4.2) [22]. The significant differences were observed at *p* < 0.05. The means which were significant were separated using Duncan’s multiple range tests.

(1) Yij=μ+Gi+eij,

where *Y*_ij_ = observation (BW, AFE, HHP, and egg quality traits) of the *i* ^th^ genotype on the *j* ^th^ pen, *µ* = overall mean, *G*_*i*_ = fixed effect of the *j* ^th^ genetic group, and *e*_ij_ = random residual error.

Diallel analysis was performed only when significant differences were detected among the genotypes. The analysis was conducted using the fixed‐effects model of Griffing [23], specifically Model I, Method I, which is a widely used approach for estimating the genetic potential of breeding lines and evaluating general and specific combining abilities in diallel crossing experiments. The effect of each parental genotype was assumed to be fixed. The statistical model used was

(2) Yij=μ+Gi+Gj+Sij+rij+eij,

where *Y*_ij_ = the observed performance of the *i* ^th^ genotype crossed with the *j* ^th^ genotype, *μ* = the overall mean of the population, *G*_*i*_ (*G*_*j*_) = GCA effect of the *i* ^th^ and *j* ^th^ parents, *S*_ij_ = SCA effect for the cross between the *i* ^th^ and *j* ^th^ parents, *r*_ij_ = reciprocal effect involving the reciprocal crosses between the *i* ^th^ and *j* ^th^ parents, and *e*_*i* *j*_ = random residual error.

## 3. Results

The results on the effect of genotype on BW and egg production traits are shown in Table 2. According to the findings, crossbreds LB × PK had heavier BW weights, followed by PK × LB. The crossbreds LB × PK had the highest AFE, followed by PK × LB, PK, and LB, respectively.

**TABLE 2:** Effect of genotype on body weight and egg production traits.

Genotypes	BW (g)	AFE (weeks)	HHP (egg/hen)
LB × LB	1463.53 ± 18.45^c^	18 ± 0.00^d^	50 ± 0.00^a^
PK × PK	1480.73 ± 17.28^c^	20 ± 0.00^c^	43.06 ± 0.22^b^
PK × LB	1567.66 ± 25.69^b^	22 ± 0.00^b^	40.91 ± 0.53^c^
LB × PK	1765.31 ± 44.77^a^	24 ± 0.00^a^	39.64 ± 0.41^d^
*p* value	< 0.0001	< 0.0001	< 0.0001

*Note:* Means in the same column with different superscripts are significant (*p* < 0.05).

Abbreviations: AFE, age at first egg; BW, body weight; HHP, hen housed production; LB, Lohmann Brown; PK, Potchefstroom Koekoek.

Table 3 presents the findings of the effect of genotype on external egg quality traits. The results indicated that LB had an influence on EW, followed by crossbred LB × PK. PK outperformed other genotypes on EL, with other genotypes showing no difference. There was no significant difference between the purebreds and crossbreds on ED; however, LB and crossbreds outperformed PK on SW.

**TABLE 3:** Effect of genotype on external egg quality traits.

Genotypes	EW (g)	EL (mm)	ED (mm)	SW(g)	SPI	SS (cm^3^)	US (cm^3^)	SR	EV (cm^3^)
LB × LB	48.44 ± 0.6^a^	51.5 ± 0.41^b^	40.68 ± 0.17^bc^	6.03 ± 0.07^a^	79.42 ± 0.49^a^	73.08 ± 0.67^a^	0.08 ± 0.00^b^	12.52 ± 0.13^c^	45.01 ± 0.72^b^
PK × PK	43.79 ± 0.22^c^	53.55 ± 0.31^a^	41.8 ± 0.36^a^	5.39 ± 0.06^b^	78.04 ± 0.47^b^	67.84 ± 0.25^c^	0.08 ± 0.00^b^	12.33 ± 0.12^c^	49.92 ± 1.16^a^
PK × LB	43.22 ± 0.13^c^	51.69 ± 0.37^b^	39.83 ± 0.41^c^	6.03 ± 0.02^a^	76.91 ± 0.2^b^	67.19 ± 0.16^c^	0.09 ± 0.00^a^	13.96 ± 0.02^a^	44.44 ± 1.71^b^
LB × PK	45.64 ± 0.21^b^	51.34 ± 0.5^b^	41.12 ± 0.43^ab^	5.91 ± 0.06^a^	80.34 ± 0.56^a^	69.98 ± 0.25^b^	0.08 ± 0.00^c^	12.95 ± 0.12^b^	47.17 ± 1.91^ab^
*p* value	< 0.0001	< 0.0001	< 0.0001	< 0.0001	< 0.0001	< 0.0001	< 0.0001	< 0.0001	0.031

*Note:* US, unit surface area shell weight; SS, shell surface area; SPI, shape index; Means in the same column with different superscripts are significant (*p* < 0.05).

Abbreviations: AFE, age at first egg; BW, body weight; ED, egg diameter; EL, egg length; EV, egg volume; EW, egg weight; HHP, hen housed production; LB, Lohmann Brown; PK, Potchefstroom Koekoek; SR, shell ratio; SW, shell weight.

The effect of genotype on internal egg quality traits is presented in Table 4. The results indicated that LB had an influence on AW and AR. The PK had a similar influence to the crossbreds on YR and Y/A. The purebreds and crossbreds had no significant differences in YW.

**TABLE 4:** Effect of genotype on internal egg quality traits.

Genotypes	AW (g)	YW (g)	AR (%)	YR (%)	Y/A
LB × LB	30.32 ± 0.42^a^	12.1 ± 0.26^ab^	62.58 ± 0.42^a^	24.9 ± 0.37^b^	40.54 ± 0.93^b^
PK × PK	26.27 ± 0.19^c^	12.12 ± 0.09^ab^	59.94 ± 0.23^b^	27.74 ± 0.22^a^	46.54 ± 0.56^a^
PK × LB	25.47 ± 0.07^d^	11.71 ± 0.05^b^	58.94 ± 0.05^c^	27.1 ± 0.05^a^	45.98 ± 0.12^a^
LB × PK	27.39 ± 0.2^b^	12.33 ± 0.13^a^	59.98 ± 0.27^b^	27.06 ± 0.28^a^	45.46 ± 0.65^a^
*p* value	< 0.0001	0.034	< 0.0001	< 0.0001	< 0.0001

*Note:* Y/A, yolk weight/albumen weight. Means in the same column with different superscripts are significant (*p* < 0.05).

Abbreviations: AR, albumen ratio; AW, albumen weight; LB, Lohmann Brown; PK, Potchefstroom Koekoek; YR, yolk ratio; YW, yolk weight.

The combined analysis of variation with mean squares and significant tests for BW, AFE, and HHP is shown in Table 5. Significant GCA effects were observed for AFE and HHP, and nonsignificant for BW. The SCA had a significant effect on AFE and HHP. The reciprocal had an influence on AFE. The GCA/SCA ratio was 0.15 for the hen used production.

**TABLE 5:** Summary of mean squares and significant tests of combining abilities for body weight, age at first egg, and hen housed production.

Source of variation	BW (g)	AFE (weeks)	HHP (egg/hen)
General combining ability	−6030.72^ns^	0.50^∗∗∗^	5.35^∗∗^
Specific combining ability	13504.21^ns^	16.00^∗∗∗^	36.49^∗∗∗^
Reciprocal	−2369.17^ns^	1.00^∗∗∗^	−0.91^ns^
General combining ability/specific combining ability	−0.45	0.03	0.15

Abbreviations: AFE, age at first egg; BW, body weight; HHP, hen housed production; ns, not significant.

^∗^Significant at *p* < 0.05.

^∗∗^ *p* < 0.01.

^∗∗∗^ *p* < 0.001.

Table 6 presents mean squares and significant tests of combining abilities for external egg quality traits. The results revealed that GCA, SCA, and reciprocal had no effect on the external egg quality traits. The study further reported a GCA/SCA ratio of less than 0.26.

**TABLE 6:** Summary of mean squares and significant tests of combining abilities for external egg quality traits.

Source of variation	EW(g)	EL (mm)	ED (mm)	SW (g)	SPI	SS (cm^3^)	US (cm^3^)	SR	EV (cm^3^)
General combining ability (GCA)	1.57^ns^	−1.16^ns^	−1.17^ns^	0.02^ns^	−1.89^ns^	1.98^ns^	−3.43^ns^	−0.11^ns^	−18.75^ns^
Specific combining ability (SCA)	−1.70^ns^	−5.75^ns^	−4.72^ns^	−0.05^ns^	−8.49^ns^	−2.27^ns^	1.82^ns^	0.61^ns^	−84.31^ns^
Reciprocal	−0.81^ns^	−3.36^ns^	−2.24^ns^	−0.06^ns^	−1.31^ns^	−0.94^ns^	−2.04^ns^	0.02^ns^	−41.67^ns^
GCA/SCA	−0.92	0.20	0.25	−0.33	0.22	−0.87	−1.88	−0.18	0.22

*Note:* SPI, shape index; SS, shell surface area; US, unit surface area shell weight.

Abbreviations: ED, egg diameter; EL, egg length; EV, egg volume; EW, egg weight; ns, not significant; SR, shell ratio; SW, shell weight.

^∗^Significant at *p* < 0.05.

^∗∗^ *p* < 0.01.

^∗∗∗^ *p* < 0.001.

Table 7 presents mean squares and significant tests of combining abilities for internal egg quality traits. The results revealed that GCA, SCA, and reciprocal had no effect on the external egg quality traits. The study further reported a GCA/SCA ratio of greater than 5.85.

**TABLE 7:** Summary of mean squares and significant tests of combining abilities for internal egg quality traits.

Source of variation	AW (g)	YW (g)	AR (%)	YR (%)	Y/A
General combining ability (GCA)	1.41^ns^	−0.23^ns^	0.07^ns^	0.32^ns^	0.32^ns^
Specific combining ability (SCA)	0.91^ns^	−0.93^ns^	0.01^ns^	−2.17^ns^	−11.97^ns^
Reciprocal	−0.35^ns^	−0.37^ns^	−1.34^ns^	−1.37^ns^	−8.30^ns^
GCA/SCA	1.54	0.25	5.89	−0.15	−0.03

*Note:* Y/A, yolk weight/albumen weight.

Abbreviations: AR, albumen ratio; AW, albumen weight; ns, not significant; YR, yolk ratio; YW, yolk weight.

^∗^Significant at *p* < 0.05.

^∗∗^ *p* < 0.01.

^∗∗∗^ *p* < 0.001.

Table 8 presents the combining abilities for BW, AFE, and HHP. The findings indicated a positive GCA on BW and AFE for PK. Meanwhile, a positive GCA was identified on HHP for LB. The results further revealed a positive SCA on BW and AFE. The reciprocal was positive for AFE.

**TABLE 8:** Combining abilities for body weight, age at first egg, and hen housed production.

Traits			
Parameters	BW (g)	AFE (weeks)	HHP (egg/hen)
*General combining abilities*			
Lohmann Brown (LB)	−4.30	−0.5	1.7
Potchefstroom Koekoek (PK)	4.30	0.5	−1.73

*Specific combining ability*			
Lohmann Brown (LB) x Potchefstroom Koekoek (PK)	98.82	2.0	−3.12
Reciprocal	97.18	1.00	−0.63

Abbreviations: AFE, age at first egg; BW, body weight; HHP, hen housed production.

The GCA, SCA, and reciprocal for external egg quality traits are shown in Table 9. There was a positive GCA for EW, SW, SPI, SS, US, and SR for LB. PK showed a positive GCA for EL, ED, and EV. The results reported a positive SCA for SW, US, and SR. The reciprocal reported negative results for all the traits except for SR.

**TABLE 9:** Combining abilities for external egg quality traits.

Traits									
Parameters	EW (g)	EL (mm)	ED (mm)	SW (g)	SPI	SS (cm^3^)	US (cm^3^)	SR	EV (cm^3^)
*General combining abilities*									
Lohmann Brown (LB)	1.16	−0.51	−0.28	0.16	0.35	1.31	0.001	0.05	−1.23
Potchefstroom Koekoek (PK)	−1.16	0.51	0.28	−0.16	−0.35	−1.31	−0.001	−0.05	1.23

*Specific combining ability*									
Lohmann Brown (LB) x Potchefstroom Koekoek (PK)	−0.84	−0.50	−0.38	0.13	−0.05	−0.94	0.003	0.52	−0.83
Reciprocal	−0.81	−3.36	−2.24	−0.06	−1.31	−0.94	−2.05	0.02	1.36

*Note:* G, general combining ability; S, specific combining ability; SS, shell surface area; SPI, shape index; US, unit surface area.

Abbreviations: ED, egg diameter; EL, egg length; EV, egg volume; EW, egg weight; LB, Lohmann Brown; PK, Potchefstroom Koekoek; SR, shell ratio; SW, shell weight.

The combining abilities for internal egg quality traits are presented in Table 10. The GCA on LB reported positive results for AW and AR and reported negative GCA for all other traits. There was a positive GCA reported for YW, YR, and Y/A for PK. There was a negative SCA for AW, YW, and AR. The results reported a positive SCA for YR and Y/A. The reciprocal reported negative results for all the internal egg quality traits.

**TABLE 10:** Combining abilities for internal egg quality traits.

Traits					
Parameters	AW (g)	YW(g)	AR (%)	YR (%)	Y/A
*General combining abilities*					
Lohmann Brown (LB)	1.01	−0.01	0.66	−0.71	−1.50
Potchefstroom Koekoek (PK)	−1.01	0.01	−0.66	0.71	1.50

*Specific combining ability*					
Lohmann Brown (LB) x Potchefstroom Koekoek (PK)	−0.93	−0.04	−0.89	0.38	1.09
Reciprocal	−0.35	−0.37	−1.34	−1.37	−8.30

*Note:* G, general combining ability; S, specific combining ability; Y/A, yolk weight/albumen weight.

Abbreviations: AR, albumen ratio; AW, albumen weight; LB, Lohmann Brown; PK, Potchefstroom Koekoek; YR, yolk ratio; YW, yolk weight.

## 4. Discussion

Crossbreeding involves improving desirable traits by mating individuals with different genetic backgrounds to produce offspring that perform better than their parents [6]. The GCA and SCA are concepts used to evaluate the performance of different breeds, strains, and lines in crossbreeding [24]. Therefore, the GCA and SCA of PK and LB chicken were evaluated.

The results of the effect of genotype on BW indicated significant differences between the purebreds and crossbreds. LB × PK had the superior BW, followed by PK × PK, PK, and LB. The outcome suggests that using LB as a sire and PK as a dam could improve BW. Similarly, the report of Teshome et al. [24] indicated that the crossbred between Sasso and PK outperformed all the purebreds on BW in a 4 × 4 diallel cross. Although the crossbreds reported the highest for AFE, this put them as the low performers, as this indicates late maturity [25]. The results implied that the crossbreds are not ideal for improving AFE. This differs from the study of Soliman et al. [13], which reported that the cross of Lohmann White and Alexandria local strain lay significantly earlier than the local strain. The difference may be due to the breeds used and the environmental effect. The findings of Soliman et al. [13] support the importance of conserving the local breeds as they serve as an important source of local genetic merits which are well adapted to harsh environments and disease resistance. Although the *F*_1_ generation may perform better than indigenous chickens, extensive crossbreeding may threaten the survival of indigenous populations through genetic erosion and the dilution of locally adapted genetic resources [2, 26]. LB had the highest number of eggs produced, followed by PK. The study of El‐Tahawy and Habashy [27] reported that LB outperformed Sinai and their crossbreds. The findings further indicate that the genotypes differed significantly in internal and external egg quality traits. Although this difference may be due to breed and breed‐by‐environment interaction, LB had an influence on EW and AW, whereas PK had an influence on EL and ED. The crossbreds influenced SW and YW; however, they did not outperform the purebreds. The finding suggests the use of purebreds for egg quality traits. This differs from the report of Maman et al. [28], which indicated that the genotypes from crossbreeding local cocks (Magia) with Isa Brown hybrid hens did not affect the egg quality traits. The findings are similar to the study of Negash et al. [11], which indicated the effect of genotype on most internal (YW, AW, and Y/A) and all external quality traits from crossing Fayoumi with Koekoek and White Leghorn.

The findings of the current study revealed that GCAs had an influence on AFE and HHP, but no influence on BW. The findings indicate that both the parent lines contribute the additive gene for AFE and HHP. They further reported a positive GCA on BW and AFE for PK, indicating the contribution of additive genetics of PK for AFE and BW. Therefore, the use of PK as a parent to exploit additive genetic variation on BW would be useful; however, the additive genetic variation on AFE would mean hens will lay eggs at a later stage [29]. The current findings on combining abilities are in harmony with the findings of Amin [30], which reported heavier pullet of Sasso chickens and their reciprocal with the Italian cross. In addition, Isaac et al. [31] reported that normal feathered local chickens × Isa Brown recorded the highest significant BW at the first egg. Although there was no significant influence of GCAs on external and internal egg quality traits, GCAs estimate of LB was positive for EW, SW, SPI, surface area, and unit surface area, and PK had a positive additive effect on EL, ED, and EV, whereas GCAs for internal egg quality traits reported a positive estimate for AW and AR for LB and positive estimate on YW and YR for PK. The finding suggests LB contributed additive genetics for most of the external and internal egg quality traits. The findings on crossbreeding of Isa Brown and frizzle‐feathered Naked Neck and normal‐feathered local chickens reported an improved EW on Isa Brown and normal‐feathered, which are in line with the current study [31]. The results of Negash et al. [11] differ from the current study, where GCA influenced external and internal egg quality traits, with positive and high values in Koekoek. GCAs are numerical values that indicate the influence of breed on its crossbred progeny [32].

The findings of the GCAs did not give information about how well the specific combination of the parents performed for a specific trait. Hence, the study also investigated the SCAs. The SCAs showed that LB and PK had a positive effect on AFE and HHP. The outcome implies that the crossbreds from LB and PK will have a delayed sexual maturity and lower hen housed egg production than their parent line, which is a reflection of the nonadditive gene action [33]. The findings are in harmony with the results of Amin [30], which reported a positive SCA for egg production traits with Italian × Mandarah and Mandarah × Italian, giving higher estimates. The SCAs estimate for external egg quality traits was negative for EW and EL and AW and YW on internal egg quality traits. The results suggest poor specific performance on the crosses between LB and PK for egg quality traits. The findings differ from the findings of Negash et al. [11], where all internal and all external egg quality traits were significant except EL, with Fayoumi × White Leghorn exhibiting positive and higher values, but Fayoumi × Koekoek had negative and lower SCAs.

The SCAs measure the average superiority or inferiority of the crosses relative to the average performance of their parental breeds [10, 24]. The results of the combining abilities did not give information about the combining ability ratio. Hence, the study also determined the combining ability ratio. The combining abilities ratio gives insights into the contribution of additive and nonadditive genetic effects [11]. The results showed a very low ratio for BW and EW, indicating that dominance or epistatic interaction is more significant than additive effects, making selection or crossbreeding ideal for improvement. However, the AR was the highest, indicating the additive effect, making pure selection highly effective. The current study disagrees with the findings of Teshome et al. [15], which reported a dominance or epistatic interaction on AFE and additive effects on EW at first egg. The study also investigated the reciprocal effects on crossbreeding, and the results showed that the reciprocal effect did not show any influence on external and internal egg quality traits; however, it was positive on AFE. This suggests a limited maternal or paternal influence on internal and external egg quality traits. The finding disagrees with the report of Teshome et al. [15], which reported that the reciprocal effect influenced all egg production traits except AFE. Soliman et al. [13] reported similar results, where a reciprocal effect for egg numbers, EW, and egg mass was not significant. The reciprocal effect reflects on the performance of offspring as the result of cross‐direction (sire vs. dam) and can indicate the maternal, paternal, and sex‐linked genetic factors [34].

## 5. Conclusion

The findings of the study revealed that there was a significant effect of genotype on egg production and egg quality traits. BW could be improved using the LB sire and PK dam. All the crossbreds increased the AFE, which resulted from the high BW in crossbreds, which is negatively related to laying performance. The results also showed significant variation due to GCA and SCA, suggesting that the additive and nonadditive variances are important for egg quality traits to be inherited. Although the results showed that the purebred outperformed the crossbreds on egg quality traits. The genetic improvement of the genotype used would be possible using selection and crossbreeding or a combination of the two. This study was conducted on two breeds; therefore, further studies are recommended to explore similar traits, including other breeds.

## Funding

The work was funded by the National Research Foundation (NRF) (Grant no. PMDS22062929025).

## Conflicts of Interest

The authors declare no conflicts of interest.

## Data Availability

The data that support the findings of this study are available from the corresponding author upon reasonable request.
